# Leveraging bipolar effect to enhance transverse thermoelectricity in semimetal Mg_2_Pb for cryogenic heat pumping

**DOI:** 10.1038/s41467-021-24161-1

**Published:** 2021-06-22

**Authors:** Zhiwei Chen, Xinyue Zhang, Jie Ren, Zezhu Zeng, Yue Chen, Jian He, Lidong Chen, Yanzhong Pei

**Affiliations:** 1 Interdisciplinary Materials Research Center, School of Materials Science and Engineering, Tongji University, Shanghai, China; 2 Center for Phononics and Thermal Energy Science, Shanghai Key Laboratory of Special Artificial Microstructure Materials and Technology, School of Physics Science and Engineering, Tongji University, Shanghai, China; 3 Department of Mechanical Engineering, The University of Hong Kong, Hong Kong SAR, China; 4 Department of Physics and Astronomy, Clemson University, Clemson, SC USA; 5 State Key Laboratory of High Performance Ceramics and Superfine Microstructure, Shanghai Institute of Ceramics, Chinese Academy of Sciences, Shanghai, China

**Keywords:** Thermoelectric devices and materials, Thermoelectrics

## Abstract

Toward high-performance thermoelectric energy conversion, the electrons and holes must work jointly like two wheels of a cart: if not longitudinally, then transversely. The bipolar effect — the main performance restriction in the traditional longitudinal thermoelectricity, can be manipulated to be a performance enhancer in the transverse thermoelectricity. Here, we demonstrate this idea in semimetal Mg_2_Pb. At 30 K, a giant transverse thermoelectric power factor as high as 400 μWcm^−1^K^−2^ is achieved, a 3 orders-of-magnitude enhancement than the longitudinal configuration. The resultant specific heat pumping power is ~ 1 Wg^−1^, higher than those of existing techniques at 10~100 K. A large number of semimetals and narrow-gap semiconductors making poor longitudinal thermoelectrics due to severe bipolar effect are thus revived to fill the conspicuous gap of thermoelectric materials for solid-state applications.

## Abstract (web summary)

Heat pumping is in high demand at cryogenic temperature, but whether thermoelectricity can take on cryogenic heat pumping is an open question. Here, the authors answer this question by leveraging bipolar effect to enhance transverse thermoelectricity in semimetal Mg_2_Pb for cryogenic heat pumping.

## Introduction

Heat management via heat pumping is critical in diverse technical and engineering fields—the operation of bolometer in space missions, industrial liquefiers, and the temperature stabilization of delicate sensors and lasering apparatuses to name a few. The compressors remain to date the mainstream commercialized technique for heat pumping^1^. The compressor-based heat pumps work efficiently only at temperatures near the gas–liquid phase transition critical point of the refrigerant used. The use of gas/liquid refrigerants and mechanical moving parts not only adds technical complexity but also poses a long-term reliability concern. Notably, there are fewer feasible refrigerants at lower temperatures.

The solid-state heat pumping is the alternative option. To this end, the refrigerant-free solid-state calorics^2^ such as magnetocaloric^3^, electrocaloric^4^, and elastocaloric^5^ techniques have attracted increasing attention. Compared to the calorics, thermoelectricity has a list of technical merits: all solid-state without moving parts, external alternating fields or mechanically moving heat exchangers^6^, responsiveness, miniaturization-friendly, no greenhouse emissions and nearly maintenance-free, all of which are due to that the working media of thermoelectricity are electrons and holes.

**Fig. 1:**
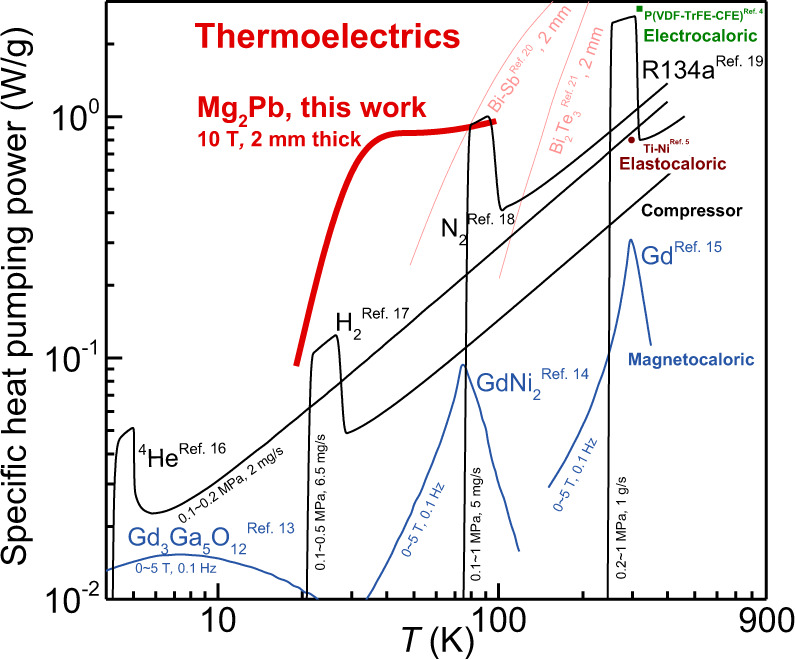
Temperature (*T*) dependent specific heat pumping power (*q*) for a variety of heat pumping techniques. The specific heat pumping power is estimated by the thermodynamic parameters of materials and the typical working conditions^4,5,37–45^ (labeled beside the solid lines). For thermoelectrics, *q* = *T*^2^*S*^2^*σ*/2/(*h*/*A*), where *h* and *A* represent the thickness along the direction of heat flow and the cross-sectional area of the materials. More details are given in Supplementary Table 1.

As shown in Fig. 1 and Supplementary Fig. 1, heat pumping is in high demand at liquid nitrogen temperature and below, where feasible refrigerants and solid-state heat pumping options barely exist. A grand open technical question is, *can thermoelectricity take on heat pumping between 10 and 100* *Kelvin?* In this work, we partially answer this question by the change of operation mode and of the choice of material.

**Fig. 2:**
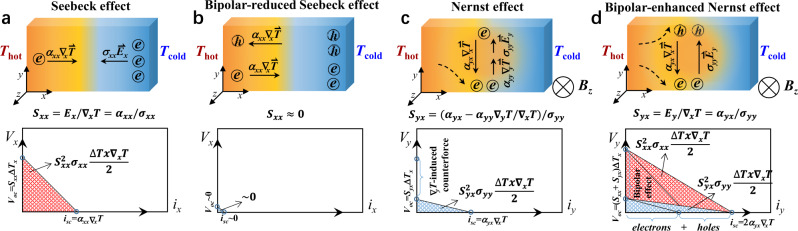
Bipolar effect on thermoelectricity. Schematic of Seebeck (**a**, **b**) and Nernst (**c**, **d**) effects without (**a**, **c)** and with (**b**, **d**) bipolar effect, from perspectives of internal current flow (top) and electric power output (bottom). The half of the product of the short-circuit current (*i*_*sc*_ = *α*_*xx*_∇_*x*_*T*, where *α* is the Seebeck conductivity) and open-circuit voltage (*V*_*oc*_ = *S*_*xx*_Δ*T*_*x*_, where *S* is the Seebeck conductivity. In longitudinal mode, *S* equals to *α* divided by electrical conductivity *σ*) represents the maximal electric power output measured by the power factor (*PF*), as indicated by the shaded areas. The bipolar-enhanced *PF* in (**d**) will be the sum of the twofold Seebeck-*PF* in (**a**) and the twofold Nernst-*PF* in (**c**). Note that (**d**) depicts the ideal case of a full compensation of thermal Hall effect for arbitrary materials, which corresponds to a perfectly symmetric conduction and valence bands with the Fermi level locating at the middle.

Effective thermoelectric heat pumping entails high heat pumping power, which is largely reduced to a high power factor (*PF* = *Sα* = *S*^2^*σ*) of the material used. The *S* is the Seebeck coefficient, *α* is the Seebeck conductivity, and *σ* is the electrical conductivity (Fig. 2a). Developing high power factor thermoelectric materials between 10 and 100 K is a challenge. The state-of-the-art thermoelectric materials such as Bi_2_Te_3_^7,8^ and PbTe^9,10^ alloys have a *S*^2^*σ* of no greater than 60 μW/cm-K^2^. This limits the existing thermoelectric materials to effectively pump out heat under typical working conditions with a specific heat pumping power of ~1 Wg^−1^ at temperatures below 100 K (Fig. 1). By comparison, the helium compressor-based heat pump enables a specific heat pumping power of ~0.2 Wg^−1^ at liquid nitrogen temperature with a rapid decay at lower temperatures. Though cryogenic heat pumping is less cost sensitive than power generation, particularly for niche applications^11,12^, there is a pressing need for high-performance cryogenic thermoelectric materials, operation modes beyond the traditional longitudinal thermoelectricity, or both.

In view of the “10-*k*_B_*T* bandgap” rule by Mahan and Sofo^13^, narrow bandgap semiconductors are the materials of choice for traditional longitudinal thermoelectric applications at cryogenic temperatures^14^. However, the bipolar effect^15^, which is inherent to narrow-gap semiconductors and severer in semimetals, is the major performance barrier. In the longitudinal mode, it is the temperature gradient that drives both electrons and holes to move in the same direction^8^. There are two effects in parallel: (i) the charge compensation between the electrons and holes that degrades the total Seebeck coefficient (*S*_*xx*_), Seebeck conductivity (*α*_*xx*_) and thus the power factor (*PF*), and (ii) the partial currents of electrons and holes due to temperature gradient (∇_*x*_*T*) induce additional Peltier heat flows and thus increases the thermal conductivity (Fig. 2b).

In the transverse mode, transverse-thermoelectricity can be enhanced by the magnetic field, which differentiates the transverse motion of charge carriers by their sign and velocity. The bipolar effect remains but acts distinctly from the case of longitudinal mode (Fig. 2 and Supplementary Fig. 2). Here, the electrons and holes carry both charge and heat to the opposite transverse direction under a magnetic field, yielding transverse electric field (*E*_*y*_) and temperature gradient (∇_*y*_*T*). In the context of linear response analyses^15^, the transverse thermoelectric current (*α*_*yx*_∇_*x*_*T*) is counterbalanced by two components to reach a steady state as illustrated in Fig. 2c: (i) (*α*_*yy*_∇_*y*_*T*), the charge current driven by the transverse temperature gradient, which is directly related to the thermal Hall effect; and (ii) (*σ*_*yy*_*E*_*y*_), the charge current driven by the Nernst effect, which at the core of electric power output and heat pumping in this work. To maximize the Nernst field *E*_*y*_, one needs to reduce the (*α*_*yy*_∇_*y*_*T*) term. To this end, the bipolar effect is a natural option. In the ideal case (Fig. 2d), the (*α*_*yy*_∇_*y*_*T*) term is gone due to a perfect compensation of electrons and holes in the thermal Hall effect. The (*σ*_*yy*_*E*_*y*_) term alone counterbalances the (*α*_*yx*_∇_*x*_*T*) term, thereby maximizing the *E*_*y*_. Therefore, the enhancement of transverse power factor is essentially an increase in short-circuit current (*α*_*yx*_∇_*x*_*T*) in a bipolar system. Hence, semimetals are promising candidates for bipolar-enhanced transverse thermoelectricity due to the abundance of both electrons and holes.

Here, we demonstrate the feasibility of transverse thermoelectric heat pumping in the cubic structured semimetal Mg_2_Pb. The specific heat pumping power is found to be superior to those of existing techniques between 10 K and liquid nitrogen temperature (Fig. 1). Note that topological Weyl or Dirac semimetals^16–21^ can be used for transverse thermoelectrics, but the working principles are distinct from the “bipolar” approach here.

## Results

### Crystal structure

**Fig. 3:**
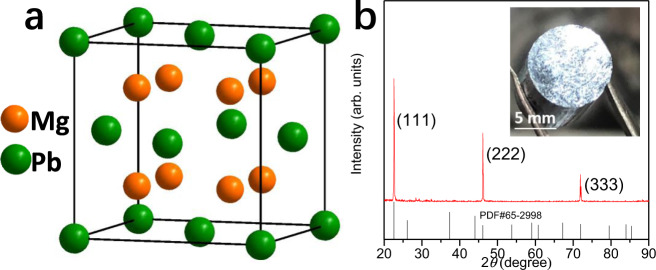
Characterization of Mg_2_Pb. **a** Crystal structure of Mg_2_Pb. **b** X-ray diffraction pattern to the cleavage surface of the (111) plane of the Mg_2_Pb crystals. Inset: a typical photograph.

Mg_2_Pb crystallizes in an anti-fluorite structure (Fig. 3a). The as-grown crystals of Mg_2_Pb in this work are usually cleaved along the (111) plane (Fig. 3b, inset shows an optical photograph). The thickness and length/width directions of the specimen are aligned parallelly and perpendicularly to the [111] direction, denoted as *z* and *x*/*y*, respectively. The crystal quality, actual composition, dominant carrier type, and the corresponding transport properties have sample dependence (more details of the as-grown samples are shown in Supplementary Fig. 3 and Supplementary Table 2), which is mainly due to the different growth conditions and the initial compositions. Hereafter, we mainly focus on the nominal composition of Mg_2.33_Pb as following.

### Band structure

**Fig. 4:**
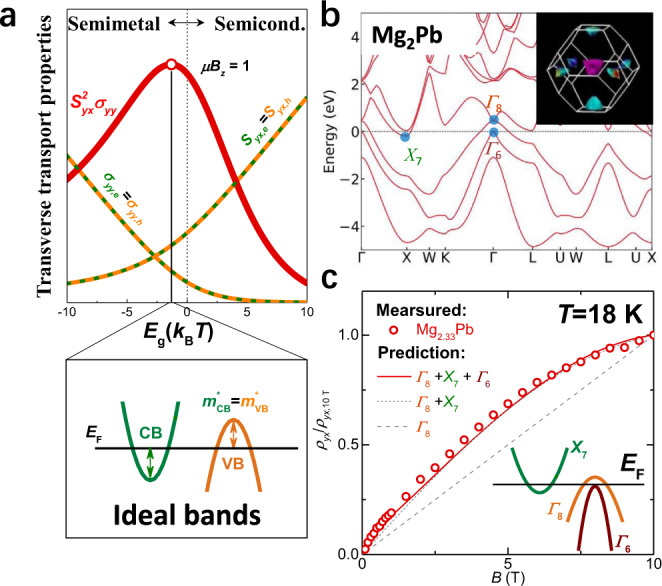
Band structures for the idea case and for Mg_2_Pb. **a** Schematic of ideal band structure for bipolar enhanced transverse thermoelectric properties as a function of band gap (*Eg*). **b** Calculated band structure and the corresponding Fermi surface for Mg_2_Pb. **c** Normalized Hall resistivity (*ρ*_*yx*_/*ρ*_*yx*,10 T_) with a three-band model prediction by the least-square fitting. The decrease in slope of *ρ*_*yx*_ vs. *B* at high fields indicates one valence band and one conduction band with comparable carrier mobilities and concentrations, and the increase of the slope at low fields indicates the involvement of an additional valence band having a higher carrer mobility and lower concentration.

Toward best performance of transverse thermoelectrics, maximal bipolar effect is favored. Hence, it is ideal that the partial *S*^2^*σ* of electrons and holes are both maximized, which corresponds to a slightly positive reduced Fermi level^22^ for both types of carriers. In other words, the ideal electronic band structure has slightly overlapped conduction (CB) and valence (VB) bands between their edges (Fig. 4a). Moreover, to facilitate a stronger magnetic field response—a high band degeneracy^23^ and a low band effective mass^24^ for each transporting band are favored. Operating at low temperatures benefits the carrier mobility (*μ*) as the carrier scattering is inhibited, which in turn enhances the transverse thermoelectric performance at a low magnetic field (*B*_*z*_).

Per our calculations and the literature^25^, the electronic band structure of semimetal Mg_2_Pb nearly meets the above guiding principle of good transverse thermoelectrics utilizing the bipolar effect. Specifically, the bands participating in charge transport include one *Γ*_8_ and one *Γ*_6_ valence band as well as one *X*_7_ conduction band in Fig. 4b. These band features are supported by the measurements of the magnetic field dependent Hall resistivity in this work (Fig. 4c) and the quantum oscillation measurements^26^. Experimentally, a control of either initial compositions or growth conditions helps locate at a nearly identical contribution of electrons and holes for a maximal bipolar effect (Supplementary Fig. 3 and Supplementary Figs. 6–7).

### Transport properties

**Fig. 5:**
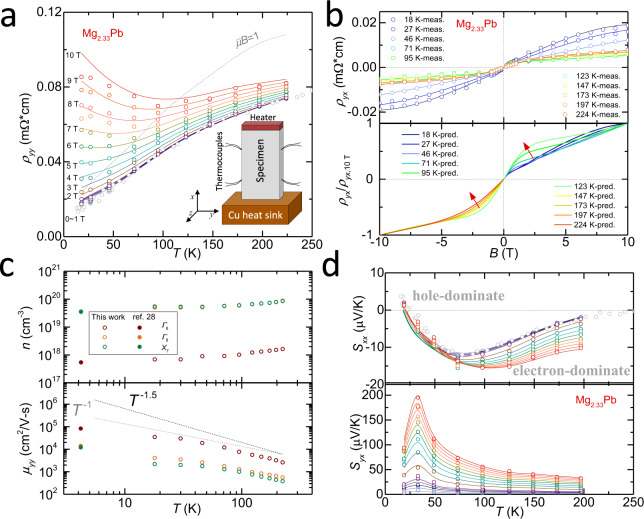
Transport properties. Temperature (*T*) and field (*B*) dependent transport properties for Mg_2.33_Pb along with the model predictions. **a** Resistivity (*ρ*_*yy*_), inset: the measurement setup. **b** Hall resistivity (*ρ*_*yx*_), the evolution of the low-field features with increasing temperature is indicated by the red arrows. **c** The estimated carrier concentration (*n*) and mobility (*μ*) for individual band, with a comparison to the literature results^26^. **d** Longitudinal (*S*_*xx*_) and transverse (*S*_*yx*_) thermopower.

The metallic *T*-dependence of resistivity (*ρ*_*yy*_) at low fields turns into an insulating one at high field (Fig. 5a), due to the gradual fulfilment of the condition of *μB*_*z*_»1 for a system with a charge compensation. The evolution of *ρ*_*yx*_ (Fig. 5b) shows that the effect of *Γ*_6_ band becomes the most noticeable at 123 K then gets damping when temperature rises continually (as indicated by the red arrows). From the resistivity and Hall resistivity, one can estimate the carrier concentration and carrier mobility based on the multi-band model. The carrier concentration increases with increasing temperature due to thermal excitation (Fig. 5c, top panel), and the carrier mobility follows a *T*^−1.5^ tendency at high temperatures or a *T*^−1^ tendency at low temperatures, indicating the scattering of carriers are mainly dominated by acoustic phonons (Fig. 5b, bottom panel). Note here, a high-quality single crystal enables a higher carrier mobility^26^.

Since the transporting conduction and valence bands are not always symmetric for Mg_2_Pb, the dominant carrier type might convert between electrons and holes at different temperatures and/or magnetic fields, which can be roughly indicated by the sign change of longitudinal thermopower (*S*_*xx*_, Fig. 5d, top panel). Overall, the absolute value of *S*_*xx*_ is rather small due to the charge compensation, in which *S*_*xx*_ is dominated by holes at low temperatures (*T* < 30 K) and by electrons at relatively high temperatures (*T* > 30 K). Qualitatively, the contributions of electrons and holes to *S*_*xx*_ become nearly identical at 30 K. This leads the partial conductivities of electrons and holes to be nearly identical, meaning the strongest bipolar effect. Hence, the transverse thermopower (*S*_*yx*_) maximizes at 30 K (Fig. 5b, bottom panel). The corresponding temperature- and field-dependent longitudinal and transverse power factors are shown in Supplementary Fig. 8.

### Modeling

To better understand the contribution of the bipolar effect on the transverse thermoelectricity of Mg_2.33_Pb particularly at 30 K, a multi-isotropic-band model is developed to analyze the results. The total conductivity tensor is expressed as^27^,

1 $$\hat{\sigma }={\sum }_{i}^{n}\frac{{n}_{i}e{\mu }_{i}}{1-{\boldsymbol{i}}{\mu }_{i}{B}_{z}}={\sum }_{i}^{n}\frac{{\sigma }_{i}}{1-{\boldsymbol{i}}{\mu }_{i}{B}_{z}}$$

where *n*_*i*_ and *μ*_*i*_ are the carrier concentration and mobility of the *i*^th^ band. The electrical conductivity (*σ*_*xx*_) and Hall conductivity (*σ*_*yx*_) are, respectively, the real and imaginary parts of conductivity tensor, while the resistivity (*ρ*_*xx*_) and Hall resistivity (*ρ*_*yx*_) are the real and imaginary parts of the inverse of conductivity tensor, as shown in Supplementary Equation (1) and Supplementary Equation (2). Similarly, the total Seebeck conductivity tensor is,

2 $$\hat{\alpha }={\sum }_{i}^{n}\frac{{S}_{{ixx}}{\sigma }_{i}+{\boldsymbol{i}}{S}_{{iyx}}{\sigma }_{i}}{1-{\boldsymbol{i}}{\mu }_{i}{B}_{z}}$$

where *S*_*ixx*_ and *S*_*iyx*_ are, respectively, the diagonal and off-diagonal thermopower of the *i*^th^ band, as shown in Supplementary Equation (5) and Supplementary Equation (6). The measured longitudinal (*S*_*xx*_) and transverse thermopower (*S*_*yx*_) are, respectively, the real and imaginary parts of the inverse of [*ασ* ^−1^], as shown in Supplementary Equation (3) and Supplementary Equation (4). Based on above equations, one can estimate the contribution of each band and the contribution of bipolar effect to the longitudinal/transverse transport properties with *n*_*i*_, *μ*_*i*_, *S*_*ixx*_, and *S*_*iyx*_ as fitting parameters.

**Fig. 6:**
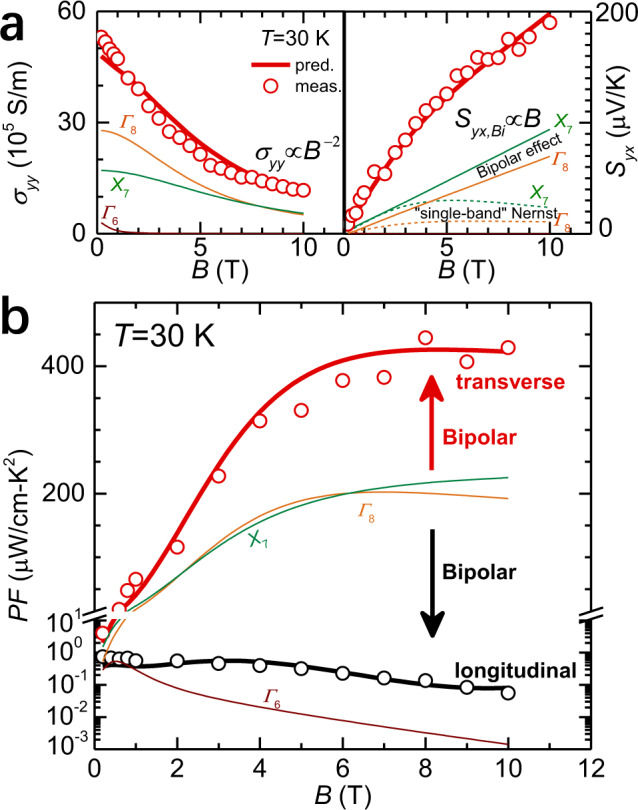
Field-dependent transport properties for Mg_2.33_Pb at 30 K. **a** Longitudinal conductivity (*σ*_*yy*_) and transverse thermopower (*S*_*yx*_). **b** Longitudinal (*S*_*xx*_^2^*σ*_*xx*_, red line) and transverse (*S*_*yx*_^2^*σ*_*yy*_, black line) power factor. The partial contributions of *Γ*_6_ and *Γ*_8_ valence bands and *X*_7_ conduction band are estimated based on a three-band model.

The total conductivity (*σ*_*yy*_) is the summation of all partial conductivities of transporting bands, and is reduced with increasing magnetic field due to the increased scattering of carriers (Fig. 6a). The total transverse thermopower (*S*_*yx*_) consists of contributions from both the “single-band” Nernst effect (Fig. 2c) and bipolar effect (Fig. 2d). The former approaches zero at high fields, while the latter is proportional to *B*_*z*_ without a saturation (Supplementary Equations (5–8)). It can be seen that the bipolar effect dominates *S*_*yx*_ particularly at high fields. Note that *Γ*_8_ valence band and *X*_7_ conduction band dominate the charge transport while *Γ*_6_ valence band barely contributes to.

The high carrier mobilities (*μ*) at liquid-nitrogen temperature or below fulfill the high magnetic field condition (*μB*_*z*_»1) within 10 T. The field-enhanced bipolar thermopower (*S*_*yx*_ is proportional to *B*, Supplementary Equation (7)) and the field-suppressed conductivity (*σ*_*yy*_ is inversely proportional to *B*^2^, Supplementary Equation (8)) lead to a transverse power factor (*S*^2^_*yx*_*σ*_*yy*_) to saturate at a certain field (Fig. 6b). Once a nearly identical partial conductivities by electrons and holes are obtained (at about a zero longitudinal thermopower) at ~30 K, the bipolar effect gets maximized thus peaks the transverse power factor (Supplementary Fig. 6). Though we highlight the results measured at 30 K, the bipolar effect actually enhances the transverse power factor and cooling power in a broad temperature range from 10 to 100 K, as shown in Fig. 1 and Supplementary Figs. 8–9.

### Power factor

**Fig. 7:**
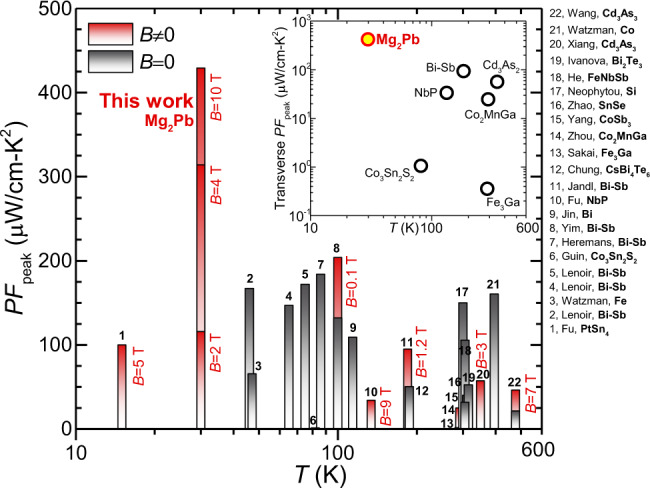
Peak power factors. Peak power factor (*PF*_peak_) of Mg_2_Pb, as compared to literature results of either longitudinal or transverse thermoelectrics^18–20,29,42,45–53^ with (red bars) or without (black bars) magnetic fields. The inset compares the transverse *PF*_peak_ for typical semiconductors and semimetals.

The transverse power factor of Mg_2_Pb is found to be orders of magnitude higher than the longitudinal counterpart at saturation fields (Fig. 6b), yielding ultra-high power factors by the standard of either longitudinal or transverse thermoelectrics (Fig. 7). It should be noted that any deviations from the optimal band structure, including largely asymmetric conduction and valence bands in InSb^28^, over-overlapped band gap in Bi–Sb^29^ and high residual carrier concentration in Pb_1-*x*_Sn_*x*_Te^30^, would lead to a decrease in transverse power factor. Fortunately, the absolute transverse power factor (>70 μWcm^−1^K^−2^) remains sufficiently high up to the liquid-nitrogen temperature (Supplementary Fig. 8). The bipolar transverse effect in semimetal Mg_2_Pb operating in the Ettingshausen configuration pumps heat much more powerfully than any existing techniques over the temperature range between 10 K and liquid nitrogen temperature (Fig. 1).

## Discussion

From the longitudinal Peltier configuration to the transverse Ettingshausen configuration with the assistance of magnetic field, this work turns the otherwise detrimental bipolar effect, ubiquitous in semimetals and narrow bandgap semiconductors, from a thermoelectric performance killer in the former to an enhancer in the latter. The jointly working electrons and holes in the Ettingshausen configuration in semimetal Mg_2_Pb lead to a much higher specific heat pumping power than any existing techniques from 10 K to liquid-nitrogen temperature. Equally importantly, these results hold promise in reviving a large number of semimetals and narrow bandgap semiconductors that exhibit as poor longitudinal thermoelectrics due to severe bipolar effect in emergent thermoelectric applications.

## Methods

### Synthesis

Mg_2_Pb forms peritectically, according to the Mg-Pb phase diagram^31^. Here, Mg_2_Pb was grown using a vertical Bridgeman technique with excess of Mg as the flux. Considering the high vapor pressure of Mg, Mg-excess were used to compensate the loss during the synthesis, with different levels for tuning the carrier concentrations. The reaction container was designed to be double-crucible, using graphite crucible as the inner lining and stainless-steel crucible as the outer one (Supplementary Fig. 4). High purity elemental Mg (99.95%) and Pb (99.99%) pieces were loaded into the crucible, which was then sealed with an arc-melting system in argon. The double-layer crucible was then sealed in a quartz ampoule. The ampoule was heated up to 650 °C, held for 15 h for pre-melting, and then cooled down to 580 °C. After a temperature stabilizing, the ampoule was pulled down at a speed of ~0.6 mm/h across a gradient temperature zone of 38 K/cm (Supplementary Fig. 4). Mg_2_Pb ingot with a diameter of ~10 mm and a length of ~40 mm was obtained.

### Characterization and calculation

X-ray diffraction (XRD, DX2000) was carried out on the cleavage surfaces to identify the crystal orientations. The band structure and Fermi surface of Mg_2_Pb were calculated based on density functional theory with VASP^32^. The generalized gradient approximation^33^ was applied and the spin–orbit coupling effects were considered. The primitive cell of Mg_2_Pb was fully relaxed before electronic structure calculations. A Γ-centered Monkhorst-Pack *k*-point mesh of 18 × 18 × 18 was used for self-consistent calculations. An energy cutoff of 400 eV and a convergence criterion of 10^−6^ eV were applied to compute the band structure. XCrySDen^34^ was used to generate the Fermi surface.

### Transport property measurements

Resistivity, thermopower, and thermal conductivity were measured simultaneously using a cryogenic magnet system (Teslatron PT, Oxford Instrument) from 8 to 300 K with a magnetic field from −10 to +10 T. The dimensions of all specimens are about 1 × 5 × 9 mm^3^. The specimen was thermally anchored to the Cu cold sink with a GE varnish. Longitudinal/transverse voltages and temperatures were measured by Cu leads and T-type thermocouples (0.003 inch in diameter with insulation layer) attached with silver epcoxy. Since Mg_2_Pb is reactive in moist air, the specimen was prepared, stored, and wired in a glovebox filled with argon and was covered by nail polish for transfer. The resistivity tensors (magneto-resistivity and Hall resistivity) were measured using a DC Montgomery method^35^ and DC Van der Pauw method^36^. Thermopower tensors (Seebeck coefficient and Nernst coefficient) were obtained from the slope of the thermopower voltage vs. temperature difference within 0–3 K, where the temperature difference was established by passing different currents to a thin film resistor attached to the top of the specimen (Supplementary Fig. 5). The thermopowers were all corrected by subtracting the absolute Seebeck coefficient of leads (copper). Thermal conductivity was measured by using the steady state method under a high vacuum (<10^−5^ Pa), where the input power was measured by monitoring the current and voltage of the thin film heater and the temperature difference was measured by longitudinally separated T-type thermocouples. Radiative heat exchange was minimized by the use of three radiation shields.

## Supplementary information

Supplementary Information

## Data Availability

The data that support the findings of this study are available from the corresponding author upon request.
