# Supplementary material for: Leveraging bipolar effect to enhance transverse thermoelectricity in semimetal Mg2Pb for cryogenic heat pumping
Source: Nat Commun. 2021 Jun 22;12:3837. doi: 10.1038/s41467-021-24161-1 (PMC8219662; doi:10.1038/s41467-021-24161-1)
Supplement: Supplementary file 1 — Supplementary Information [file 41467_2021_24161_MOESM1_ESM.pdf]

Supplementary information

Leveraging bipolar effect to enhance transverse thermoelectricity in semimetal Mg_2Pb for cryogenic heat pumping

Zhiwei Chen[1,2], Xinyue Zhang[1], Jie Ren[2], Zezhu Zeng[3], Yue Chen[3], Jian He[4], Lidong Chen[5,*] and Yanzhong Pei[1,*]

[1]Interdisciplinary Materials Research Center, School of Materials Science and Engineering, Tongji Univ., 4800 Caoan Rd., Shanghai, 201804, China.

[2]Center for Phononics and Thermal Energy Science, Shanghai Key Laboratory of Special Artificial Microstructure Materials and Technology, School of Physics Science and Engineering, Tongji University, 200092 Shanghai, P. R. China.

[3]Department of Mechanical Engineering, The University of Hong Kong, Pokfulam Road, Hong Kong SAR, China.

[4]Department of Physics and Astronomy, Clemson University, Clemson, SC 29634-0978, USA.

[5]State Key Laboratory of High Performance Ceramics and Superfine Microstructure, Shanghai Institute of Ceramics, Chinese Academy of Sciences, Shanghai 200050, China.

[*]Email: cld@mail.sic.ac.cn (LC); yanzhong@tongji.edu.cn (YP)

Section 1: Specific heat pumping power for various techniques

The heat pumping power depends not only on the thermodynamic parameters of materials, but also on the kinetics of working process. Broadly, the gross pumping power is proportional to the rate of thermodynamic processes while the internal loss is inversely proportional to the square of this rate[1, 2, 3, 4, 5, 6] at a given external field (Supplementary Figure 1). Taking thermoelectrics as an example[7], the Peltier effect induced gross pumping power equals to the product of S,T, and I, where S is the Seebeck coefficient, T is the absolute temperature and I is the electrical current. The internal loss is due to Joule heating of $RI^2/2$ with R as the electrical resistance. Accordingly, the net heat pumping power (q_{max}) maximizes at a current of ST/R, attaining $q_{max}=T^2S^2/R/2=T^2S^2\sigma/2/(h/A)$. Here, σ, h and A represent the electrical conductivity, the thickness along the direction of heat flow, and the cross-sectional area of the thermoelectric materials, respectively. In this work, we use a typical thickness of h=2 mm of commercial thermoelectric devices to estimate the heat pumping power. Similar analyses have been done for compressors and magnetocalorics without taking into account the internal energy losses (due to hysteresis losses and/or convective flow losses) as shown in Fig. S1. Note that $q_{net}\approx q_{gross}=\Delta sTf$ for magnetocalorics and $q_{net}\approx q_{gross}=\Delta sTm/M$ for compressors estimated in this work are essentially the gross heat pumping power under typical working conditions, where Δs is isothermal entropy change and f is operating frequency for magnetocalorics; m is mass flow rate and M is refrigerant charge for compressors. The detailed maximum specific cooling power and working conditions for different technologies are listed in Supplementary Table 1.

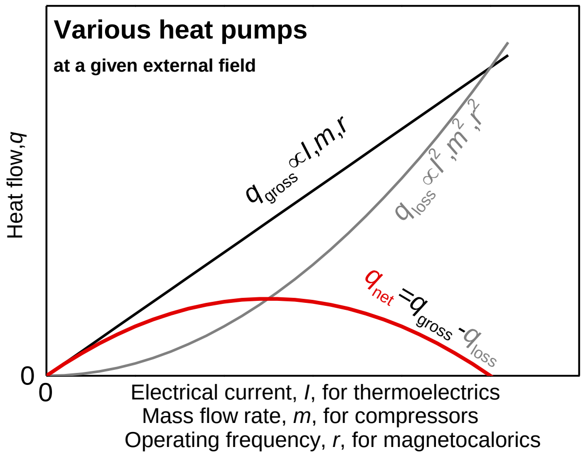

Supplementary Figure 1. The underlying similarities in terms of thermodynamics for thermoelectrics, compressors and magnetocalorics, which involve the net heat pumping power (q_{net}), the gross heat pumping power (q_{gross}) and the internal losses (q_{lose}) at various working conditions such as current (I) for thermoelectrics, the mass flow rate (m) for compressors and operating frequency (r) for magnetocalorics. In this work, q_{lose} are not included for the estimation of q_{net} for compressors and magnetocalorics.

Supplementary Table 1. The specific heat pumping power for thermoelectrics, magnetocalorics and compressors within 4 to 100 K.

**Techniques**	**Materials**	**Max Specific heating pumping power (W/g)**	**Field (T or GPa or MV/cm)**	**Operating condition (A or mg/s or Hz)**	**Ref.**
Thermoelectric	Mg_2Pb	#0.96 @ 98 K	10 T	#3.9 A	This work
	$PtSn_4$	#0.029 @ 15K	5 T	#2.9 A	[8]
	NbP	#0.24 @ 84 K	9 T	#0.98 A	[9]
	Cd_3As_2	#0.013 @ 100 K	3 T	#0.27 A	[10]
	Bi-Sb	#0.014 @ 97 K	1.2 T	#0.22 A	[11]
	URu_2Si_2	#0.00094 @ 7.5 K	6 T	#0.28 A	[12]
	$Co_3Sn_2S_2$	#0.00082 @ 82K	N/A	#0.095 A	[13]
	$CsBi_4Te_6$	#0.54 @ 100 K	N/A	#1.3 A	[14]
	Bi_2Te_3	#0.68 @ 100 K	N/A	#2.0 A	[15]
	Bi-Sb	#1.3 @ 92 K	N/A	#4.6 A	[16]
	Co_3Sb	#0.19 @ 96 K	N/A	#0.89 A	[17]
Magnetocaloric	$Dy_3Ga_5O_{12}$	*9.4×10^{-4} @ 4.5 K	5.1 T	*0.2 Hz	[18]
	$Gd_2(SO_4)_3\cdot8H_2O$	*1.4×10^{-4} @ 4.2 K	2.5 T	*0.017 Hz	[3]
	$Gd_2(SO_4)_3\cdot8H_2O$	*0.0091 @ 4.2 K	5 T	*0.3 Hz	[4]
	$Gd_3Ga_5O_{12}$	*0.018 @ 4.2 K	5 T	*0.8 Hz	[19]
	$Gd_3Ga_5O_{12}$	*4.3×10^{-4} @ 20 K	6.7 T	*0.05 Hz	[20]
	$Gd_3Ga_5O_{12}$	*0.0037 @ 15 K	6 T	*0.1 Hz	[21]
	$Gd_3Ga_5O_{12}$	*0.0047 @ 15 K	4.5 T	*0.38 Hz	[22]
	$Gd_3Ga_5O_{12}$	*0.0085 @ 16 K	5 T	*0.05 Hz	[23]
	$Gd_3Ga_5O_{12}$	*0.0011 @ 4.2 K	3 T	*0.1 Hz	[24]
	$DyAl_{2.2}$	*0.0021 @ 59 K	5 T	*0.003 Hz	[25]
	$Gd_3Ga_5O_{12}$	*3.3×10^{-4} @ 10 K	5.6 T	*0.005 Hz	[26]
	$GdNi_2$	*0.0024 @ 60 K	5 T	*0.1 Hz	[27]
	$GdNi_2$	*0.0024 @ 45 K	7 T	*0.1 Hz	[27]
	$Er_xGd_{1-x}Al_2$	*0.001 @ 12 K	1 T	*0.1 Hz	[27]
	$GdNi_2$	*0.019 @ 64 K	7 T	*0.1 Hz	[28]
	$Gd_3Ga_5O_{12}$	#0.015 @ 7.5 K	5 T	#0.1 Hz	[21, 29, 30]
	$GdNi_2$	#0.095 @ 74 K	5 T	#0.1 Hz	[28, 31, 32]
Compressor	He^4	#0.29 @ 100 K	0.1-0.2 MPa	#2 mg/s	[33, 34, 35]
	H_2	#0.14 @ 100 K	0.1-0.5 MPa	#6.5 mg/s	[36, 37]
	N_2	#0.38 @ 100 K	0.1-1.0 MPa	#5 mg/s	[5, 38, 39]
	R134a	-	0.2-1.0 MPa	#1 g/s	[40, 41]

*Measurement; #Estimation.

Section 2: Multi-band modeling and maximal transverse magneto-power factor

Under a zero net heat flow, the electrical resistivity (ρ_{xx}) and Hall resistivity (ρ_{yx}) under a magnetic field (B_z) for a multi-isotropic-band transport system can respectively be formulized as[42] Supplementary Equation 1 and Equation 2:

$$\rho_{xx} = \frac{\sigma_{xx}}{\sigma_{xx}^2+\sigma_{yx}^2} = \frac{\sum_i^n \sigma_{ixx}}{(\sum_i^n \sigma_{ixx})^2 + (\sum_i^n \sigma_{iyx})^2} = \frac{\sum_i^n \frac{n_i e\mu_i}{1+\mu_i^2B_z^2}}{(\sum_i^n \frac{n_i e\mu_i}{1+\mu_i^2B_z^2})^2 + (\sum_i^n \mu_i B_z \frac{n_i e\mu_i}{1+\mu_i^2B_z^2})^2} \quad , \tag{1}$$

and

$$\rho_{yx} = \frac{-\sigma_{yx}}{\sigma_{xx}^2+\sigma_{yx}^2} = \frac{-\sum_i^n \sigma_{iyx}}{(\sum_i^n \sigma_{ixx})^2 + (\sum_i^n \sigma_{iyx})^2} = \frac{-\sum_i^n \mu_i B_z \frac{n_i e\mu_i}{1+\mu_i^2B_z^2}}{(\sum_i^n \frac{n_i e\mu_i}{1+\mu_i^2B_z^2})^2 + (\sum_i^n \mu_i B_z \frac{n_i e\mu_i}{1+\mu_i^2B_z^2})^2} \quad , \tag{2}$$

where σ_{xx} is the total electrical conductivity, σ_{yx} is the total Hall conductivity, n_i and μ_i are the carrier concentration and mobility of the ith band, elementary charge e is negative for electrons and positive for holes, and the former/latter subscript denotes the direction of the thermodynamic flow/force.

Under a zero net current flow, the total longitudinal thermopower (S_{xx}) and transverse thermopower (S_{yx}) are the function of conductivity, Hall conductivity, Seebeck conductivity (α_{xx}) and Nernst conductivity (α_{yx}). S_{xx} and S_{yx} can be written as

$$S_{xx} = \frac{\sigma_{xx}\alpha_{xx} + \sigma_{yx}\alpha_{yx}}{\sigma_{xx}^2+\sigma_{yx}^2} = \frac{1}{\sigma_{xx}^2+\sigma_{yx}^2}\left[\sum_i^n S_{iyx}\left(-\sigma_{iyx}\sigma_{xx} + \sigma_{ixx}\sigma_{yx}\right) + \sum_i^n S_{ixx}\left(\sigma_{ixx}\sigma_{xx} + \sigma_{iyx}\sigma_{yx}\right)\right] \quad , \tag{3}$$

and

$$S_{yx} = \frac{\sigma_{xx}\alpha_{yx} - \sigma_{yx}\alpha_{xx}}{\sigma_{xx}^2+\sigma_{yx}^2} = \frac{1}{\sigma_{xx}^2+\sigma_{yx}^2}\left[\sum_i^n S_{iyx}\left(\sigma_{ixx}\sigma_{xx} + \sigma_{iyx}\sigma_{yx}\right) + \sum_i^n S_{ixx}\left(\sigma_{iyx}\sigma_{xx} - \sigma_{ixx}\sigma_{yx}\right)\right] \quad . \tag{4}$$

The second terms in the bracket in Supplementary Equation 3 and Equation 4 stand for the contributions from the interactions between bands, which are termed as "the bipolar effect" for simplicity herein. In the following equations, S_{ixx} and S_{iyx} are respectively the longitudinal and transverse thermopower of the ith band. Thermopower is the energy of the current flow with respect to the Fermi level, which depends on the density of states, group velocity and relaxation time. Under the approximations of spherical energy band and the parabolic density of state, the conventional definition of thermopower[43] can be reduced to[44]:

$$S_{ixx} = \frac{k_{\mathrm{B}}}{e}\left(\frac{\langle \tau_i E_i\rangle}{\langle \tau_i\rangle} - E_{\mathrm{F},i}\right)\frac{1}{k_{\mathrm{B}}T} \quad where\ \langle \tau^p E^q\rangle = \int_0^\infty \frac{x^{1.5+q}\tau^p}{1+\mu^2B_z^2}\frac{\partial f_0}{\partial(-x)}dx, \tag{5}$$

and

$$S_{iyx} = \frac{\mu_i B_z}{1+\mu_i^2B_z^2}\frac{k_{\mathrm{B}}}{e}\left[\left(\frac{\langle \tau_i^2 E_i\rangle}{\langle \tau_i^2\rangle} - E_{\mathrm{F},i}\right) - \left(\frac{\langle \tau_i E_i\rangle}{\langle \tau_i\rangle} - E_{\mathrm{F},i}\right)\right]\frac{1}{k_{\mathrm{B}}T} \quad . \tag{6}$$

It can be seen that S_{ixx} is proportional to the energy difference between the average carrier energy weighted by the relaxation time of carriers and the Fermi level (parenthesis in Supplementary Equation 5), while S_{iyx} is proportional to the energy difference between the average energy weighted by the square of the relaxation time and the one weighted by the relaxation time (the bracketed term in Supplementary Equation 6). Noted that the second term in the bracket of Supplementary Equation 6 represents the counter-contribution of the transverse temperature gradient as shown in the Fig. 2c in the main text. The difference leads S_{ixx} and S_{iyx} to show a different Fermi level dependence and a different magnetic field dependence as shown in Supplementary Figure 2.

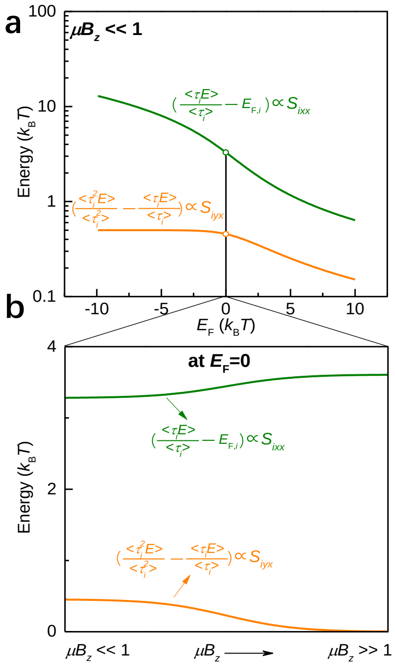

Supplementary Figure 2. The Fermi level dependent energy difference terms at a low magnetic field limit (a) and a schematic field dependent energy difference terms at E_F=0 (b) for longitudinal and transverse thermopowers. Showing that the longitudinal thermopower is usually much larger than the transverse magneto-thermopower for a single band system[7].

For simplicity yet without losing generality, a system with only one conduction band (with a subscript of e) and one valence band (with a subscript of p) are taken as an archetype to locate the conditions for maximizing the transverse magneto-power factor for a system involving a bipolar conduction. Transverse thermopower S_{yx} increases with increasing magnetic field, and at a given magnetic field S_{yx} maximizes when the partial conductivities of electrons and holes are identical (σ_e/σ_h=1). Once the high-filed condition is fulfilled (μB_z>1), it can be seen that the bipolar effect completely account for S_{yx} (Supplementary Figure 3a). With a decrease in the total conductivity under a magnetic field (Supplementary Figure 3b), transverse magneto-power factor tends to saturate starting at a magnetic field fulfilling μB_z=1 and dominated by the bipolar effect (Supplementary Figure 3c). This point towards that nearly symmetrical electron and hole transports (usually corresponds to symmetrical bands at a nearly zero total Seebeck coefficient, S_{xx}~0) and large enough magnetic fields are vital to maximize the transverse magneto-power factor.

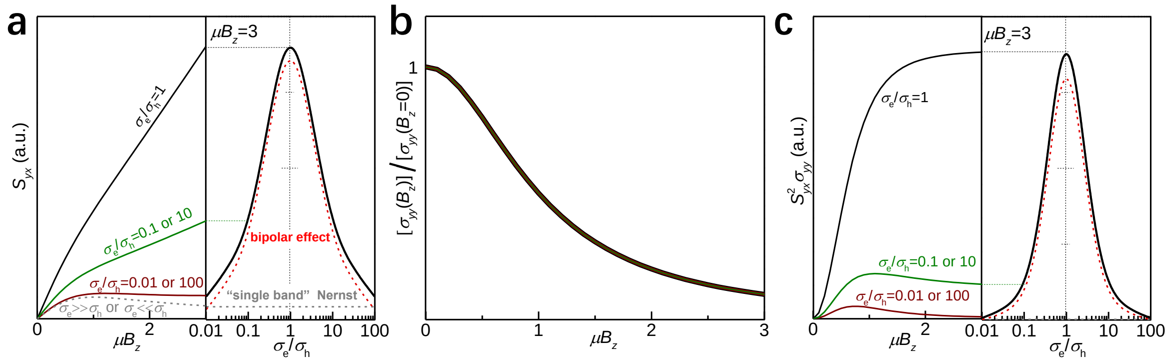

Supplementary Figure 3. The field (μB_z) and partial conductivity ratio (σ_e/σ_h) dependent transverse thermopower (a), relative electrical conductivity (b) and transverse magneto-power factor (c).

In the high-field limit (μB_z>>1) when concentrations and conductivity of electrons and holes are identical (n_e=n_h and σ_e=σ_h), the transverse thermopower has a much simpler form of Supplementary Equation 7:

$$S_{yx} = \frac{S_{e,yx}}{2} + \frac{S_{h,yx}}{2} + \frac{\mu B_z (S_{h,xx} - S_{e,xx})}{2} \quad , \tag{7}$$

and similarly the equation of resistivity reduces to Supplementary Equation 8:

$$\rho_{yy} = \frac{\sigma_{yy}}{\sigma_{yy}^2 + \sigma_{yx}^2} = \frac{\mu^2 B_z^2}{2\sigma} \quad . \tag{8}$$

Section 3: Synthesis and Methods

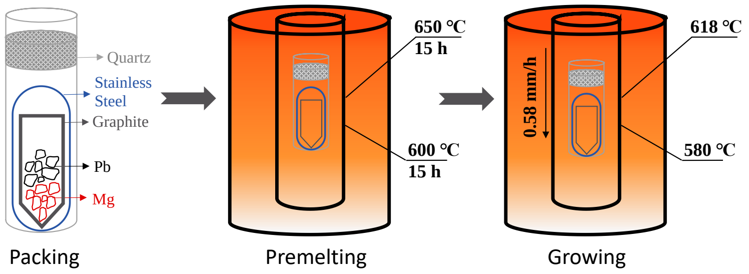

Supplementary Figure 4. Schematic diagram of crystal growth technique for Mg_2Pb.

Supplementary Table 2. The nominal composition, crystal forms, growth condition and room-temperature Seebeck coefficient (RTS) of Mg_2Pb crystals.

Nominal compositions	Crystal forms	Growth conditions	RTS (μV/K)
Mg_2Pb	Oriented crystals	Vertical Bridgeman with growth speed of 1.8 mm/h at temperature gradient of 38 K/cm	+41
$Mg_{2.05}Pb$	Oriented crystals	Vertical gradient freeze with cooling speed of 3 K/h at temperature gradient of 25 K/cm	+10
$Mg_{2.33}Pb$	Oriented crystals	Vertical Bridgeman with growth speed of 0.6 mm/h at temperature gradient of 38 K/cm	+0.4
$Mg_{2.4}Pb$	Poly crystal	Furnace cooling	+2.1

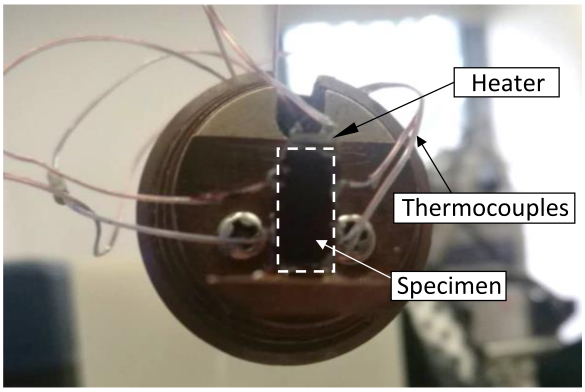

Supplementary Figure 5. Typical photograph for the sample mounting. Note here that the specimen was covered by nail polish to isolate air during the whole mounting and measurements.

It should be noted that, the measurement of thermomagnetic effects is non-trivial. The canonical thermomagnetic effects are often defined at the isothermal or at the adiabatic limit. The experimental setup shown in Supplementary Figure 6 enables us to measure the thermomagnetic effects under a condition between the isothermal and adiabatic limit[45]. In other words, the boundary conditions of measurement change over with the time of measurement from isothermal at the initial moment to adiabatic at the final moment in absence of forced heat exchange with the environment. As a caveat, the measured voltages and temperatures cannot be attributed exclusively to any single canonical thermomagnetic effect, rather, they stem from a combination of the isothermal and adiabatic thermomagnetic effects. Nonetheless, the foci of this work are the transverse thermomagnetic transport coefficients, such as the transverse magneto-thermopower (S_{yx}). It is known the difference between the isothermal and adiabatic transverse thermopowers tends to be much smaller when the longitudinal counterparts (S_{xx}) are rather small [45]. In this work, S_{xx} is intrinsically small due to the strong compensation of electrons and holes (aka the bipolar effect). Hence, the measured S_{yx} are practically close to the canonical (isothermal or adiabatic) values.

Section 4: Transport properties

Experimentally, different growth conditions and starting compositions were used to tune the partial conductivity ratio (σ_e/σ_h) and more details are given in Supplementary Section 2. Indeed, as we expect, once the electron/hole conductivity ratio approaches unity (σ_e/σ_h~1), the transverse magneto-power factor tends to maximize, leading to a power factor >400 μW/cm-K^2 at 30 K and 10 T in the sample grown with an initial composition of $Mg_{2.33}Pb$. More details on the transport properties for all the samples are shown in Supplementary Figure 6-9.

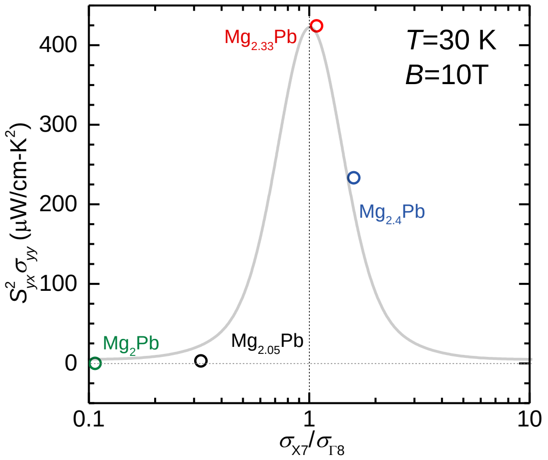

Supplementary Figure 6. The partial conductivity ratio of X_7 conduction band and Γ_8 valence band ($\sigma_{X7}/\sigma_{\Gamma 8}$) dependent transverse magneto-power factor for different initial crystal-growth compositions of Mg_2Pb.

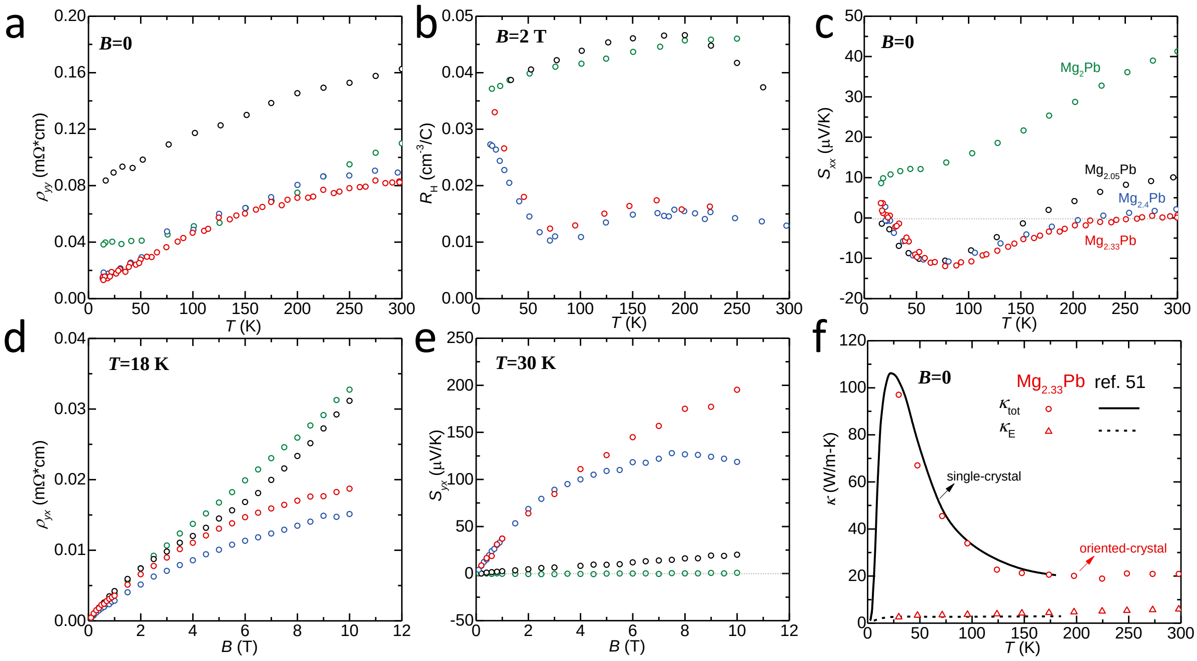

Supplementary Figure 7. Temperature and magnetic field dependent transport coefficients of samples grown from different initial compositions, with a comparison to the literature results[46]. Samples with different growth conditions and initial compositions show apparent differences in properties.

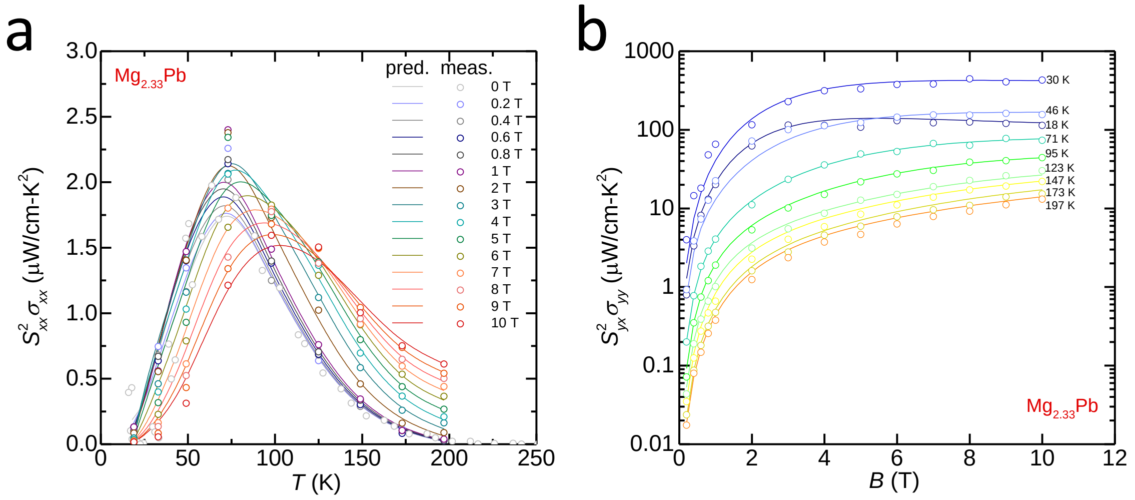

Supplementary Figure 8. Temperature and field dependent longitudinal (a) and transverse (b) power factors for $Mg_{2.33}Pb$.

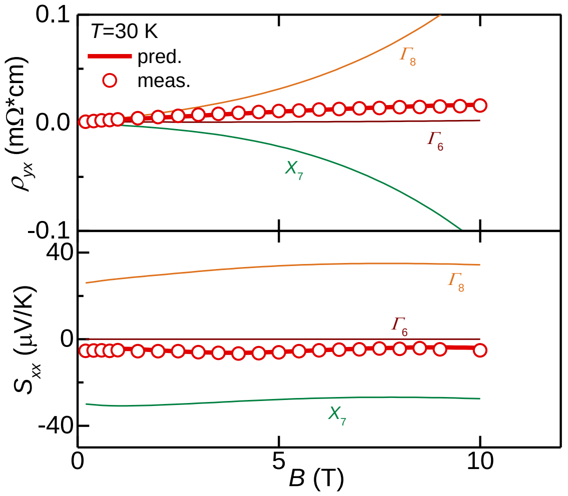

Supplementary Figure 9. The partial contributions to Hall resistivity and longitudinal thermopower.

Supplementary references:

1. DeGregoria A. J. Modeling the Active Magnetic Regenerator. In: *Advances in Cryogenic Enginnering* (Springer, New York, 1992).
2. Kral S F B. J. A. *Magnetic Refrigeration: A Large Cooling Power Cryogenic Refrigeration Technology* (Springer, Boston, 1991).
3. Barclay J. A., Moze O., Paterson L. A reciprocating magnetic refrigerator for 2–4 K operation: Initial results. *J. Appl. Phys.* **50**, 5870-5877 (1979).
4. Delpuech C B. R., Mardion G B, et al. Double acting reciprocating magnetic refrigerator: first experiments. *Cryogenics* **21**, 579-584 (1981).
5. H.J. Holland J. F. B., N. Boersma, H.J. M. ter Brake, H. Rogalla. Miniature 10–150 mW Linde-Hampson cooler with glass-tube heat exchanger operating with nitrogen *Cryogenics* **38**, 407-410 (1998).
6. Wang B., Gan Z. H. A critical review of liquid helium temperature high frequency pulse tube cryocoolers for space applications. *Progress in Aerospace Sciences* **61**, 43-70 (2013).
7. Goldsmid H. J. *Introduction to Thermoelectricity* (Springer, Heidelberg, 2009).
8. Fu C., *et al.* Largely Suppressed Magneto-Thermal Conductivity and Enhanced Magneto-Thermoelectric Properties in $PtSn_4$. *Research (Wash D C)* **2020**, 4643507 (2020).
9. Fu C., *et al.* Large Nernst power factor over a broad temperature range in polycrystalline Weyl semimetal NbP. *Energy & Environmental Science* **11**, 2813-2820 (2018).
10. Xiang J., *et al.* Large transverse thermoelectric figure of merit in a Dirac semimetal. *China-Phys. Mech. Astron.* **63**, 237011 (2020).
11. Jandl P., Birkholz U. Thermogalvanomagnetic properties of Sn-doped $Bi_{95}Sb_5$ and its application for solid state cooling. *J. Appl. Phys.* **76**, 7351 (1994).
12. Bel R., Jin H., Behnia K., Flouquet J., Lejay P. Thermoelectricity of URu_2Si_2: Giant Nernst effect in the hidden-order state. *Phys. Rev. B* **70**, 220501(R) (2004).
13. Guin S. N., *et al.* Zero-Field Nernst Effect in a Ferromagnetic Kagome-Lattice Weyl-Semimetal $Co_3Sn_2S_2$. *Adv. Mater.* **31**, e1806622 (2019).
14. Chung D. $CsBi_4Te_6$: A High-Performance Thermoelectric Material for Low-Temperature Applications. *Science* **287**, 1024-1027 (2000).
15. Ivanova L D G. Y. V. Thermoelectric Properties of Bi_2Te_3-Sb_2Te_3 Single Crystals in the Range 100-700 K *Inorganic materials* **36**, 672-677 (2000).
16. Heremans J. P., Jin H., Zheng Y., Watzman S. J., Prakash A. BiSb and spin-related thermoelectric phenomena. *Proc. SPIE* **9821**, 98210I (2016).
17. Yang J., Morelli D. T., Meisner G. P., Chen W., Dyck J. S., Uher C. Effect of Sn substituting for Sb on the low-temperature transport properties of ytterbium-filled skutterudites. *Phys. Rev. B* **67**, 165207 (2003).
18. Numazawa T., Kimura H., Sato M., Maeda H. Carnot Magnetic Refrigerator Operating between 1.4 K and 10 K. *Cryogenics* **33**, 547-554 (1993).
19. Lacaze A. F., Beranger, R., Mardion, G. B., Claudet, G., Lacaze, A. A. Double acting reciprocating magnetic refrigerator: recent improvements. In: *Advances in Cryogenic Engineering* (Springer, New York, 1984).
20. H. Nakagome N. T. H., H. Ogiwara, T. Numazawa, Y. Watanabe, T. Hashimoto. The Helium Magnetic Refrigerator I: Development and Experimental Results. In: *Advances in Cryogenic Engineering* (Springer, Boston, 1984).
21. Barclay J. A., et al. Experimental results on a low-temperature magnetic refrigerator. In: *Advances in cryogenic engineering* (Springer, New York, 1986).
22. Nakagome H. K. T., Ogiwara H., Fujita T., Yazawa T., Hashimoto T. Reciprocating Magnetic Refrigerator for Helium Liquefaction. In: *Advances in Cryogenic Engineering* (Springer, New York, 1986).
23. Numazawa T. H. T., Nakagome H. . Improvement of Liquefaction Efficiency of the Heat Pipe Type Magnetic Refrigerator. In: *Advances in Cryogenic Engineering* (Springer, New York, 1986).
24. Hashimoto T. Recent Investigations on Refrigerants for Magnetic Refrigerators. In: *Advances in Cryogenic Engineering*

Materials (Springer, New York, 1986).
25. Matsumoto K., Ito, T., & Hashimoto, T. An Ericsson Magnetic Refrigerator for Low Temperature. In: *Advances in Cryogenic Engineering* (Springer, New York, 1988).
26. Numazawa T. H. T., Nakagome H., Tanji N., Horigami O. . The Helium Magnetic Refrigerator II: Liquefaction Process and Efficiency. In: *Advances in Cryogenic Engineering* (Springer, New York, 1984).
27. DeGregoria A. J., Feuling, L. J., Laatsch, J. F., Rowe, J. R., Trueblood, J. R., Wang, A. A. Test results of an active magnetic regenerative refrigerator. In: *Advances in Cryogenic Engineering* (Springer, New York, 1992).
28. Wang A. A., Johnson, J. W., Niemi, R. W., Sternberg, A. A., Zimm, C. B. Experimental Results of an Efficient Active Magnetic Regenerator Refrigerator. In: *Cryocoolers 8* (Springer, New York, 1995).
29. Wikus P., Canavan, E., Heine, S. T., Matsumoto, K., & Numazawa, T. Magnetocaloric Materials and the Optimization of Cooling Power Density. *Cryogenics* **62**, 150-162 (2014).
30. McMichael R. D., Ritter J. J., Shull R. D. Enhanced magnetocaloric effect in $Gd_3Ga_{5-x}Fe_xO_{12}$. *J. Appl. Phys.* **73**, 6946-6948 (1993).
31. Plaza E. J. R., *et al.* A comparative study of the magnetocaloric effect in RNi_2 (R=Nd,Gd,Tb) intermetallic compounds. *J. Appl. Phys.* **105**, 013903 (2009).
32. Matsumoto K., Asamato K., Nishimura Y., Zhu Y., Abe S., Numazawa T. Magnetocaloric effect of RM_2 (R= rare earth,M=Ni, Al) intermetallic compounds made by centrifugal atomization process for magnetic refrigerator. *Journal of Physics: Conference Series* **400**, 052020 (2012).
33. Carty R. D. M. Thermodynamic Properties of Helium 4 from 2 to 1500 K at Pressures to 10^8 Pa. *J. Phys. Chem. Ref. Data* **2**, 923 (1973).
34. J.D.Daunt E. L. A closed-cycle Joule-Thomson liquefier and cryostat for He^3. *Cryogenics* **10**, 476-479 (1970).
35. J.J.Bock L. D., M.Kawada, H.Matsuhara, T.Matsumoto,A.E.Lange. ^{4}He refrigerator for space. *Cryogenics* **34**, 635-640 (1994).
36. Bender E. Equation of state of normal hydrogen in the range 18 to 700 K and 1 to 500 bar. *VDI Forschungsheft N* **609**, 15-20 (1982).
37. Bhandari P., Prina M., Bowman R. C., Paine C., Pearson D., Nash A. Sorption coolers using a continuous cycle to produce 20 K for the Planck flight mission. *Cryogenics* **44**, 395-401 (2004).
38. Lemmon E. W., Jacobsen R. T., Penoncello S. G., Friend D. G. Thermodynamic properties of air and mixtures of nitrogen, argon, and oxygen from 60 to 2000 K at pressures to 2000 MPa. *J. Phys. Chem. Ref. Data* **29**, 331-385 (2000).
39. Lerou P. P. P. M., *et al.* Progress in Micro Joule-Thomson Cooling at Twente University. In: *Cryocoolers 13* (Springer, New York, 2005).
40. TillnerRoth R., Baehr, H. D. An International Standard Formulation for the Thermodynamic Properties of 1,1,1,2-Tetrafluoroethane (HFC-134a) for Temperatures from 170 K to 455 K and Pressures up to 70 MPa. *J. Phys. Chem. Ref. Data 23* **23**, 657-729 (1994).
41. Fatouh M., Kafafy M. E. Experimental evaluation of a domestic refrigerator working with LPG. *Appl. Therm. Eng.* **26**, 1593-1603 (2006).
42. Shastry B. S. Electro Thermal Transport Coefficients at Finite Frequencies. *Rep. Prog. Phys.* **72**, 016501 (2008).
43. Vargiamidis V., Thesberg M., Neophytou N. Theoretical model for the Seebeck coefficient in superlattice materials with energy relaxation. *J. Appl. Phys.* **126**, (2019).
44. Delves R. T. The prospects for Ettingshausen and Peltier cooling at low temperatures. *Br. J. Appl. Phys.* **13**, 440 (1962).
45. Franz Freibert, Timothy W. Darling, Albert Migliori, Trugman S. A. Thermomagnetic effects and measurements. In: *Semeconductors and semimetals* (Elsevier, 2002).
46. J. J. Martin H. R. S. Thermal conductivity of magnesium plumbide. *J Appl Phys* **45**, 2428 (1974).
